# How Common Is Imported Cutaneous Leishmaniasis in Romania? Two Case Reports

**DOI:** 10.3390/microorganisms13061207

**Published:** 2025-05-25

**Authors:** Victoria Birlutiu, Gabriela Iancu, Rares-Mircea Birlutiu, Simin Aysel Florescu

**Affiliations:** 1 Faculty of Medicine, Lucian Blaga University of Sibiu, 550169 Sibiu, Romania; victoria.birlutiu@ulbsibiu.ro; 2 Infectious Diseases Clinic, County Clinical Emergency Hospital, 550245 Sibiu, Romania; 3 Clinic of Dermatology, County Emergency Hospital Sibiu, 550012 Sibiu, Romania; 4 Faculty of Medicine, Department 14-Orthopedics, Anaesthesia Intensive Care Unit, “Carol Davila” University of Medicine and Pharmacy, 050474 Bucharest, Romania; 5 Foisor Clinical Hospital of Orthopedics, Traumatology, and Osteoarticular TB, 021382 Bucharest, Romania; 6 “Dr. Victor Babes” Clinical Hospital of Infectious and Tropical Diseases, “Carol Davila” University of Medicine and Pharmacy, 050474 Bucharest, Romania

**Keywords:** cutaneous leishmaniasis, Romania, case report

## Abstract

Background: Leishmaniasis is a vector-borne zoonotic disease caused by protozoa of the genus *Leishmania*. While it is endemic in the Mediterranean Basin and the Balkans, Romania remains a non-endemic country. However, climate change, increased international travel, and the documented presence of competent vectors (*Phlebotomus* spp.) have raised concerns about the potential emergence of autochthonous cases. Case Presentation: We report two cases of imported cutaneous leishmaniasis (CL) diagnosed in central Romania, a region without previously confirmed human or animal cases. The first case involved a 31-year-old male with a recent travel history to Spain, presenting with erythematous papules and plaques that evolved into ulcerated lesions. The diagnosis was confirmed histopathologically and by a PCR. Treatment with miltefosine was effective, with minimal hepatic toxicity and a sustained response at a six-month follow-up. The second case concerned an 11-year-old boy who had traveled to Elba, Italy. He developed ulcerative lesions that progressed rapidly and were complicated by *Pseudomonas aeruginosa* superinfection. Despite an initially negative smear, PCR testing of the skin lesion confirmed the presence of CL. Antifungal therapy with fluconazole led to clinical improvement; treatment was ongoing at the time of publication. Discussion: These cases highlight the diagnostic and therapeutic challenges associated with CL in non-endemic settings. The varied clinical evolution underscores the importance of considering leishmaniasis in the differential diagnosis of chronic, non-healing cutaneous lesions, particularly in patients with a travel history to endemic regions. Conclusions: Increased awareness among clinicians, supported by accurate diagnostic tools and public health surveillance, is essential to identify and manage imported leishmaniasis. Given the absence of a licensed vaccine and the growing risk of vector expansion in Eastern Europe, these cases support the WHO’s inclusion of leishmaniasis among the priority neglected tropical diseases targeted for intensified global control efforts by 2030.

## 1. Introduction

Leishmaniasis is a parasitic disease classified by the WHO as a neglected tropical disease, predominantly found in tropical and subtropical regions of the globe, as well as in southern Europe. It is endemic in Asia, the Middle East, North Africa, the Mediterranean region, and Central and South America. In Romania, cases of leishmaniasis are typically imported ones, posing diagnostic challenges due to the rarity of the disease. Here, we present two cases of cutaneous leishmaniasis, highlighting the diagnostic difficulties encountered.

From a taxonomic perspective, leishmaniasis is caused by protozoa of the family *Trypanosomatidae*, order *Kinetoplastida*, and genus *Leishmania*, which includes more than 20 species [1]. The disease is transmitted through the bites of dipteran insects of the genera *Phlebotomus* and *Lutzomyia*, with over 90 known “Old World” and “New World” vector species”. Transmission can also occur, albeit rarely, through blood transfusion, the use of contaminated needles among intravenous drug users, organ transplantation, or laboratory exposure. The primary reservoirs of *Leishmania* include domestic and peridomestic animals such as ruminants and dogs, as well as wild animals like wolves, jackals, and foxes.

Cutaneous leishmaniasis (CL) is diagnosed annually in 700,000 to 1 million cases [1], with the majority occurring in the Americas, the Mediterranean Basin, Central Asia, and the Middle East. In contrast, visceral leishmaniasis (VL) is confirmed in 50,000 to 90,000 cases per year, predominantly in regions such as Brazil [2], East Africa, and India. In the absence of treatment, VL has a fatality rate of up to 95%. Clinically, CL is characterized by painless plaques at the site of the sandfly bite, which may expand and present on the face or extremities as nodular lesions, papules, or plaques. The lesions can mimic fungal infections or leprosy [3], while some cases progress to necrotic ulcerations [4].

Mucocutaneous leishmaniasis (MCL) accounts for 90% of the cases in Bolivia, Peru, Ethiopia, and Brazil. Mucosal involvement—most commonly affecting the nasal, oral, or tracheal mucosa—typically manifests as ulcerative lesions approximately 24 months after the initial cutaneous infection. This progression occurs through hematogenous or lymphatic dissemination and may lead in some cases to respiratory dysfunction [2,5].

Post-kala-azar dermal leishmaniasis (PKDL), the fourth clinical form of the disease, emerges as a sequela of visceral leishmaniasis (VL). It presents with perioral and nasal lesions that tend to extend progressively. The condition is associated with a heightened immune response, particularly involving interferon-gamma, against *Leishmania* infection [6].

*Leishmania infantum* is widely recognized as the only autochthonous species causing leishmaniasis in both humans and animals throughout Europe, with the exception of isolated instances involving *L. tropica* in certain regions of Greece and *L. donovani sensu stricto* in Cyprus. Its distribution spans across 22 European countries where both animal and human infections have been documented, including Spain and Italy, the countries in which the two cases described in our report occurred. The presence of *L. infantum* in these areas is well established and supported by a large body of epidemiological data. Between 2005 and 2020, Spain reported a total of 8260 hospital discharges due to leishmaniasis, representing 4946 patients, with 91% of those with available clinical data diagnosed with VL, 6% with CL, and 4% with MCL. The cumulative incidence over this 15-year period was 0.67 cases per 100,000 population, with notable increases in the VL and CL incidence observed in various years throughout the study period. Similarly, Italy reported 2509 hospital cases between 2011 and 2016, of which 88% involved Italian nationals and 82% were VL cases. The estimated cumulative incidence for this period was 0.70 per 100,000 population. These figures align with the known endemicity of *L. infantum* in Mediterranean Europe and reflect both the persistent transmission of the parasite and the burden it poses to public health systems. Despite some discrepancies between data from national and WHO-GHO reporting systems, the consistent identification of *L. infantum* across multiple datasets underscores its epidemiological relevance in both countries [7].

The clinical presentation of leishmaniasis is influenced by the virulence of the *Leishmania* species, the host’s immune system, and the patient’s genetic predisposition. Patients with an effective Th1-mediated primary immune response exhibit excellent control over parasitemia and typically develop the cutaneous form of the disease. In contrast, patients with a predominant Th2-mediated immune response, characterized by the synthesis of neutralizing antibodies, fail to control the intracellular replication of the parasite, leading to visceral or disseminated cutaneous leishmaniasis [8]. *Leishmania* enters the skin in a promastigote form, which, in immunocompetent individuals, is typically phagocytosed and destroyed by macrophages without causing clinical disease. If the promastigotes survive, they transform into amastigotes and can disseminate through the reticuloendothelial system. The cutaneous lesion develops at the site of the sandfly bite, initially as a papule and later forming a granuloma with a tendency to ulcerate. These lesions may spontaneously heal over 6–18 months or become chronic, with only a small proportion progressing to mucosal and mucocutaneous forms [8]. Individuals with a robust Th1-mediated immune response develop a delayed-type hypersensitivity reaction characterized by interferon-gamma (IFN-γ) production, which facilitates the clearance of intracellular parasites within dendritic cells and macrophages [9,10,11]. The initial immune response to *Leishmania major* infection is Th1-skewed and directed against a conserved glycosomal antigen expressed in both promastigotes and amastigotes, with CD4+ T cells playing a central role [12]. The persistence of cutaneous lesions despite therapy is attributed to ongoing local inflammation driven by proinflammatory cytokines and chemokines released from activated monocytes, neutrophils, and CD4+ T cells [13]. Additionally, post-treatment parasite persistence in immunological “sanctuary sites” within clinically intact skin may maintain partial protective immunity while also posing a risk of reactivation [14,15]. Long-term persistence may be due to low iNOS expression in dermal macrophages, reduced monocyte recruitment, or the accumulation of regulatory T cells and IL-10 with immunosuppressive effects [16]. Although the pathogenesis of cutaneous leishmaniasis is not fully understood, recent immunogenomic studies have suggested that treatment failure may be associated with an exaggerated type I interferon response in certain species and host immune profiles, particularly in cases unresponsive to meglumine antimoniate therapy [13].

Most patients develop symptoms between 3 and 8 months after the sandfly bite, with the reported extremes ranging from 10 days to a maximum of 34 months. In VL, the clinical presentation is characterized by prolonged febrile syndrome, splenomegaly, hepatomegaly [5,17], lymphadenopathy, progressive weight loss that in some cases can lead to cachexia, secondary anemia, and eventual organ failure within the following two years. Parasitic aggression contributes to hemolytic anemia, bone marrow invasion by infected mononuclear cells, and the splenic sequestration of erythrocytes and leukocytes, leading to hemorrhagic manifestations. Neutropenia causes a predisposition to secondary infections, while thrombocytopenia increases the risk of bleeding. Autoimmune processes have also been reported. The mortality rates vary significantly, with the highest recorded in Sudan in 1988, where 100,000 deaths were reported due to VL [17].

The diagnosis of this disease is suggested by the presence of symptoms in individuals in endemic regions or with a history of travel to such areas. In CL, the hallmark presentation is a painless, non-purulent ulcer localized on the face or extremities. In VL, prolonged fever, hepatosplenomegaly, fatigue, weight loss, anemia, and leukopenia are characteristic clinical findings. Laboratory confirmation relies on molecular testing (PCR) and the detection of *Leishmania* amastigotes in tissue samples obtained via aspiration or biopsy. Additionally, a non-invasive DNA sampling method—using tape strips from ulcerative or closed skin lesions—has been described [18]. The Montenegro intradermal skin test may also be used to support the diagnosis.

The etiological treatment of VL is administered parenterally and includes pentavalent antimonials (sodium stibogluconateand meglumine antimoniate), amphotericin formulations (deoxycholate amphotericin and liposomal amphotericin B), or pentamidine. For CL, oral therapies are more commonly employed, such as miltefosine or azole antifungals (fluconazole).

## 2. Case Presentations

### 2.1. First Case Report

**Figure 1:**
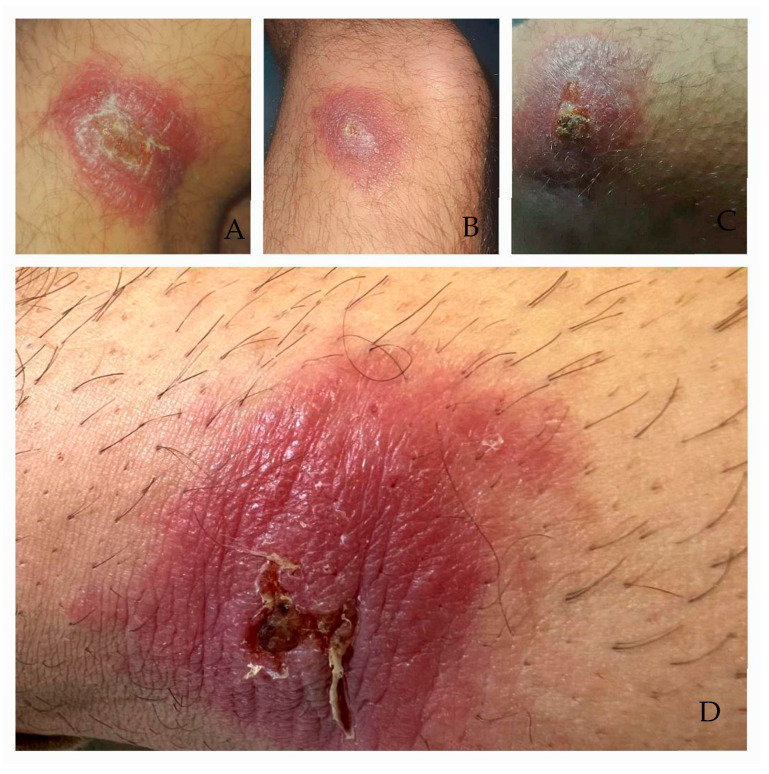
Erythematous, infiltrated plaques (**A**,**B**) and nodules © located on the lower limbs. The lesions appeared relatively well defined, though with irregular borders and a mildly edematous aspect. They ranged in size from 3 to 5 cm, with some showing central ulceration (**C**,**D**).

We report the case of a 31-year-old Caucasian male patient with no significant medical history who presented himself to our department with multiple cutaneous lesions. The initial presentation consisted of erythematous, infiltrated papuloid lesions, well circumscribed with regular margins, measuring approximately 0.5 cm in diameter. Centrally, a superficial ulceration was observed, prurigo-like in appearance. The lesions were pruritic and distributed across the upper and lower extremities, as well as the abdominal region. The patient reported a recent travel history in August 2023 to *L’Ametlla de Mar*, for tourism, which is situated in the province of Tarragona, Catalonia, on the Mediterranean coast of Spain. During his stay, he experienced an insect bite on his right lower limb while on the beach. Over the following weeks, he sought multiple dermatological consultations and was treated with topical corticosteroid creams, either alone or in combination with topical antibiotics, without clinical improvement. The lesions demonstrated slow peripheral extension over time, progressively increasing in size and infiltrating underlying structures. They evolved into erythematous, infiltrated plaques with a tendency toward central ulceration (Figure 1).

#### Histopathological Findings

After eight months of dermatological follow-ups, a skin biopsy of the left knee lesion was performed. Histopathological examination revealed morphological alterations in both the epidermis and dermis. Numerous epithelioid granulomas were observed, with a tendency toward confluence but without necrosis. These granulomas were composed of epithelioid histiocytes, multinucleated giant cells, and a diffuse lymphocytic infiltrate. The inflammatory infiltrate extended into deeper layers, demonstrating a perivascular, periadnexal, and perineural distribution. Epidermal changes included acanthosis, along with areas of ulceration. The histopathological examination concluded with a diagnosis of granulomatous dermatitis, suggesting several potential etiologies, including fungal infection, *Mycobacterium tuberculosis*/*leprosy*, cutaneous manifestations of Crohn’s disease, or sarcoidosis with integumentary involvement. Further investigations were recommended, including bacteriological examinations, periodic acid–Schiff (PAS) staining, and immunohistochemical analysis to rule out lymphomatous infiltration. Immunohistochemical staining was negative for CD4, CD8, CD3, Ki67, and CD20, effectively excluding lymphoproliferative disorders.

Following an infectious disease examination, cutaneous leishmaniasis was suspected, with the clinical suspicion subsequently confirmed by the attending dermatologist. A second skin biopsy confirmed the diagnosis, with microscopic examination revealing extracellular *Leishmania* spp. Amastigotes and molecular tests further confirming the infection. The diagnosis was established at the Victor Babeș Clinical Hospital of Infectious and Tropical Diseases in Bucharest. Although the diagnosis was confirmed by a PCR, species-level molecular typing—such as a species-specific PCR or species-specific sequencing—was unfortunately not available.

The patient underwent treatment with miltefosine at a dosage of 50 mg three times daily for 28 days. By day 21 of the therapy, liver function abnormalities were observed, with alanine aminotransferase (ALT) levels rising to 180 U/L. These abnormalities were successfully managed with hepatoprotective medication, and the liver function normalized one month after the discontinuation of miltefosine.

**Figure 2:**
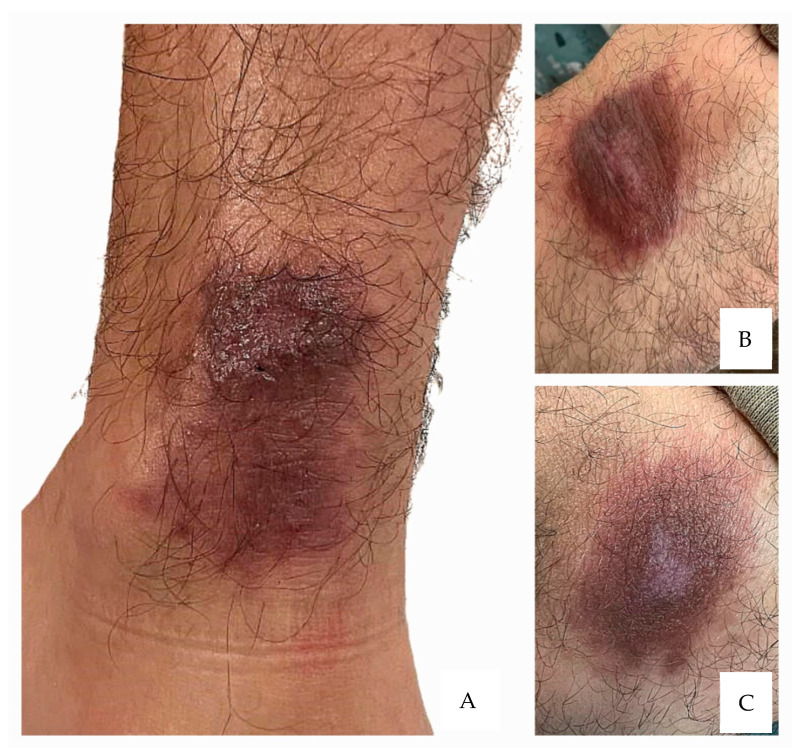
Clinical appearance of the cutaneous lesions one month after the completion of treatment. Hyperpigmented plaques with mild infiltration, likely reflecting underlying dermal fibrosis, and central scarring consistent with re-epithelialized ulcerations. The lesions remained stable in size, with no signs of local inflammation (**A**–**C**).

**Figure 3:**
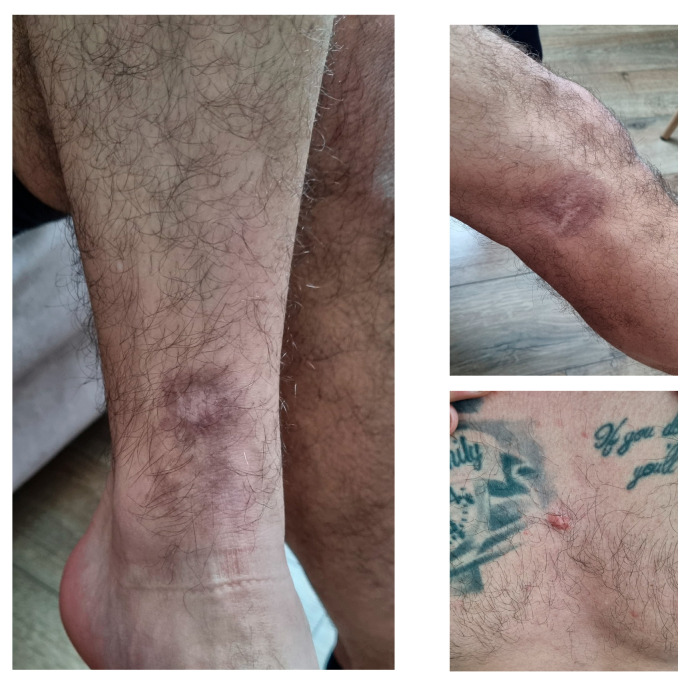
Clinical appearance of the cutaneous lesions in May 2025; photos provided by the patient.

At discharge, the lesions appeared as intensely hyperpigmented plaques, with mildly infiltrated overlying skin and a central scarred area (Figure 2). The lesions’ appearance at the beginning of May 2025 is shown in Figure 3.

### 2.2. Second Case Report

**Figure 4:**
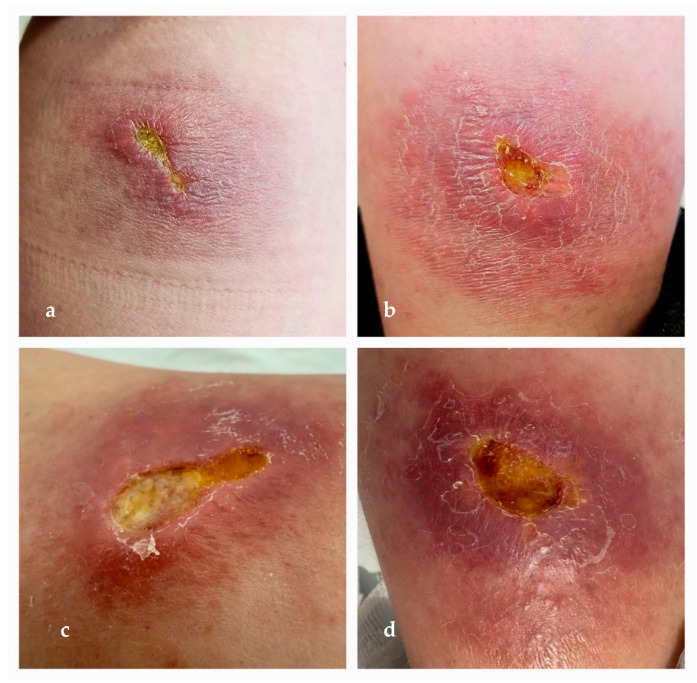
(**a**,**b**) Erythematous, indurated, and warm infiltrated plaques with poorly defined margins and progressive peripheral extension. Central areas exhibited necrotic ulcerations covered by adherent yellow fibrino-necrotic material. Clinical aspect at 8 weeks from onset. (**c**,**d**) Stabilization of infiltrated plaques, with reduction in periulcerative inflammation and partial regression of peripheral erythema, despite persistence of vasculitic-like appearance. Central ulcerations had increased in both size and depth, with continued presence of fibrino-necrotic debris. Clinical aspect at 10 weeks from onset.

The second case concerns an 11-year-old boy from Brașov County, admitted to our department in March 2025 for two ulcerative lesions on the posterior aspect of the left leg, with a two-month history of progressive enlargement and purulent discharge. Prior to admission, the patient had been evaluated by dermatology and infectious disease specialists at a local hospital, without a definitive diagnosis. Notably, the patient had traveled to the coastal area of Elba Island, which is part of the Tuscany Archipelago, Italy, an area known for its Mediterranean climate, in September 2024. In December 2024, he developed two pruritic, prurigo-like nodular lesions with a small central ulceration on the posterior left leg. Over time, the lesions gradually expanded peripherally, evolving into erythematous, infiltrated plaques that were poorly demarcated, with irregular borders, measuring approximately 3 × 5 cm. Centrally, they developed necrotic ulcerations with an ecthyma-like appearance, surrounded by an edematous, raised, and infiltrated border (March 2025). Within two weeks, the ulcers increased in both size and depth, becoming covered with adherent yellow fibrinous exudate, exhibiting sharply demarcated edges, and lacking signs of re-epithelialization. The erythematous, infiltrated plaques showed partial regression, appearing less indurated, but developed a violaceous, vasculitic-like aspect (see Figure 4a–d). The lesions were found to be secondarily infected with *Pseudomonas aeruginosa*, sensitive to meropenem and amikacin. Based on a clinical suspicion of leishmaniasis, a smear was taken from the lesion, which was negative. However, therapy with fluconazole was initiated at the insistence of the legal guardian. The patient was referred to the Victor Babeș Clinical Hospital for Infectious and Tropical Diseases in Bucharest for further evaluation, where cutaneous leishmaniasis was confirmed by a PCR; although the diagnosis was confirmed by a PCR, species-level molecular typing was unfortunately not available.

## 3. Discussion

Leishmaniasis is a parasitic zoonotic disease endemic to the Mediterranean Basin and the Balkans, with variable incidence rates. The highest incidence is reported in Greece, Italy, Spain, Albania, and Montenegro. A total of 17 countries have reported autochthonous cases, with visceral leishmaniasis being the predominant form, while cutaneous cases remain underdiagnosed. Between 2005 and 2020, the highest incidence was recorded in Albania, at 2.15 cases per 100,000 inhabitants, followed by Greece, Spain, Montenegro, Malta, and North Macedonia, with incidence rates ranging from 0.53 to 0.42 per 100,000 inhabitants. In Italy, the incidence was reported at 0.16 per 100,000, while Portugal had the lowest recorded incidence at 0.09 per 100,000 inhabitants [7]. Romania is considered a non-endemic country for *Leishmania*. However, with the ongoing climate changes, the presence of Phlebotomus spp. in certain regions [19] may facilitate the emergence of human cases. This risk is further supported by the fact that certain species, such as *Phlebotomus perfiliewi* and *P. neglectus*, are known to be competent vectors for the transmission of *Leishmania infantum* [20]. The surveillance of leishmaniasis in dogs has confirmed the presence of autochthonous cases in the southern regions of Romania, including Râmnicu Vâlcea [21] and Argeș [22]. Molecular testing for *Leishmania infantum* DNA yielded positive results in 20.1% of 149 tested dogs [22]. Additional cases have been reported in Galați [23] and Dolj [24], highlighting the need for extensive epidemiological studies and continuous surveillance.

In terms of human pathology, the first case of visceral leishmaniasis (VL) in Romania was reported by Manicatide in 1912, with an additional 24 cases diagnosed in 1954 [25]. Between 1999 and 2005, imported cases of visceral leishmaniasis (VL) were confirmed at the Clinical Hospital of Infectious and Tropical Diseases in Bucharest and Victor Babes Hospital in Timisoara, with patients originating from Greece and Spain [26]. In the following years, between 2005 and 2020, cases of VL continued to originate from Greece and Italy [27], with a total of 42 human cases diagnosed [7].

The reported cases occurred in central Romania, in a region where neither human nor animal cases of leishmaniasis had been previously confirmed. The patients had a documented travel history to Spain and Italy, respectively, and an insect bite was recalled in the first case, while in the second case there was not known contact with a possible vector. The clinical presentation differed significantly between the two patients. In the pediatric case, the lesions ulcerated rapidly and became secondarily infected with *Pseudomonas aeruginosa*, displaying resistance to cephalosporins, fluoroquinolones, and even some carbapenems (including imipenem–cilastatin). In contrast, treatment with miltefosine in the adult patient proved effective, with only minimal hepatic side effects, which resolved within one month following the completion of the therapy. At the six-month follow-up, the residual lesions remained stable, and the patient demonstrated a sustained therapeutic response, confirming the treatment efficacy and absence of drug resistance. Although hypothetical resistance to miltefosine was considered, given its potential molecular basis through the deletion of the miltefosine susceptibility locus (MSL) [28], it was not observed in this case. For our pediatric patient, treatment with fluconazole was initiated, with the progressive closure of the ulcerations observed. At the time of publication, the treatment was ongoing. Although neither case involved disseminated disease, the diagnoses are highly relevant and educational for clinicians practicing in non-endemic areas, where the rarity of such conditions can significantly hinder clinical suspicion and pose substantial challenges in differential diagnosis.

These cases underscore the importance of clinical awareness and differential diagnosis in non-endemic areas. In addition, a thorough epidemiological investigation is essential, including the careful assessment of any potential exposure events, which may have occurred up to 34 months prior to the symptom onset.

To date, there is no effective prophylactic strategy against *Leishmania* that ensures the control and eradication of the parasite. Vaccines evaluated in preclinical studies have been associated with persistent skin lesions at the inoculation site [29]. Leishmaniasis remains a critical global health priority for the World Health Organization and has been included among the 20 priority diseases targeted for prevention, control, and eradication between 2021 and 2030.

## 4. Conclusions

Although Romania is considered a non-endemic country for leishmaniasis, the two cases reported here—occurring in a central region without previously documented human or animal cases—highlight the increasing relevance of this parasitic disease beyond traditionally endemic zones. Both patients had epidemiological links to southern European countries. Their contrasting clinical evolution and therapeutic responses illustrate the heterogeneity of cutaneous leishmaniasis, particularly when complicated by bacterial superinfection or delayed diagnosis. These cases emphasize the critical need for heightened clinical suspicion, even in non-endemic areas, especially in the context of climate change, increasing international travel, and the documented presence of competent vectors in parts of Romania. Accurate and timely diagnosis, combined with effective treatment and follow-ups, are essential in preventing complications and limiting disease progression. Furthermore, the absence of a viable prophylactic strategy, alongside ongoing challenges in vaccine development, reinforces the importance of public health surveillance, physician education, and multidisciplinary collaboration in addressing leishmaniasis as a neglected tropical disease. These findings support the World Health Organization’s prioritization of leishmaniasis for intensified prevention, control, and eradication efforts by 2030.

## Data Availability

The original contributions presented in this study are included in the article. Further inquiries can be directed to the corresponding authors.

## Abbreviations

The following abbreviations are used in this manuscript:

CL	Cutaneous leishmaniasis
MCL	Mucocutaneous leishmaniasis
PKDL	Post-kala-azar dermal leishmaniasis
VL	Visceral leishmaniasis

## References

[1] World Health Organization Leishmaniasis. https://www.who.int/news-room/fact-sheets/detail/leishmaniasis.

[2] Desjeux P. (2004). Leishmaniasis: Current Situation and New Perspectives. Comp. Immunol. Microbiol. Infect. Dis..

[3] Hashiguchi Y., Gomez E.L., Kato H., Martini L.R., Velez L.N., Uezato H. (2016). Diffuse and Disseminated Cutaneous Leishmaniasis: Clinical Cases Experienced in Ecuador and a Brief Review. Trop. Med. Health.

[4] de Vries H.J.C., Schallig H.D. (2022). Cutaneous Leishmaniasis: A 2022 Updated Narrative Review into Diagnosis and Management Developments. Am. J. Clin. Dermatol..

[5] McGwire B.S., Satoskar A.R. (2014). Leishmaniasis: Clinical Syndromes and Treatment. Int. J. Med..

[6] Zijlstra E.E. (2019). Biomarkers in Post-Kala-Azar Dermal Leishmaniasis. Front. Cell. Infect. Microbiol..

[7] Maia C., Conceição C., Pereira A., Rocha R., Ortuño M., Muñoz C., Jumakanova Z., Pérez-Cutillas P., Özbel Y., Töz S. (2023). The estimated distribution of autochthonous leishmaniasis by Leishmania infantum in Europe in 2005–2020. PLoS Negl. Trop. Dis..

[8] Piccioni A., Valletta F., Zanza C., Longhitano Y., Torelli E., de Cunzo T., Esperide A., Brigida M., Ojetti V., Covino M. (2020). Rapid Clinical Management of Leishmaniasis in Emergency Department: A Case Report with Clinical Review of Recent Literature. Biology.

[9] Bacellar O., Lessa H., Schriefer A., Machado P., Ribeiro de Jesus A., Dutra W.O., Gollob K.J., Carvalho E.M. (2002). Up-regulation of Th1-type responses in mucosal leishmaniasis patients. Infect Immun..

[10] Ueha S., Shand F.H., Matsushima K. (2012). Cellular and molecular mechanisms of chronic inflammation-associated organ fibrosis. Front. Immunol..

[11] Scott P., Natovitz P., Coffman R.L., Pearce E., Sher A. (1988). Immunoregulation of cutaneous leishmaniasis. T cell lines that transfer protective immunity or exacerbation belong to different T helper subsets and respond to distinct parasite antigens. J. Exp. Med..

[12] Mou Z., Li J., Boussoffara T., Kishi H., Hamana H., Ezzati P., Hu C., Yi W., Liu D., Khadem F. (2015). Identification of broadly conserved cross-species protective Leishmania antigen and its responding CD4+ T cells. Sci. Transl. Med..

[13] Gómez M.A., Belew A.T., Vargas D., Giraldo-Parra L., Rebellón-Sanchez D., Alexander T., El Sayed N. (2025). Innate biosignature of treatment failure in human cutaneous leishmaniasis. Nat. Commun..

[14] Aebischer T. (1994). Recurrent cutaneous leishmaniasis: A role for persistent parasites?. Parasitol. Today.

[15] Okwor I., Liu D., Beverley S.M., Uzonna J.E. (2009). Inoculation of killed Leishmania major into immune mice rapidly disrupts immunity to a secondary challenge via IL-10-mediated process. Proc. Natl. Acad. Sci. USA.

[16] Stenger S., Donhauser N., Thüring H., Röllinghoff M., Bogdan C. (1996). Reactivation of latent leishmaniasis by inhibition of inducible nitric oxide synthase. J. Exp. Med..

[17] Zijlstra E.E., El-Hassan A.M. (2001). Leishmaniasis in Sudan. 3. Visceral Leishmaniasis. Trans. R. Soc. Trop. Med. Hyg..

[18] Taslimi Y., Sadeghipour P., Habibzadeh S., Mashayekhi V., Mortazavi H., Müller I., Lane M.E., Kropf P., Rafati S. (2017). A Novel Non-Invasive Diagnostic Sampling Technique for Cutaneous Leishmaniasis. PLoS Negl. Trop. Dis..

[19] Daraban Bocaneti F., Ivanescu L.M., Miron L., Tanase O.I., Dascalu M.A. (2022). An Overview on Leishmaniasis in Romania: Diagnosis and Therapeutics. Trop. Med. Infect. Dis..

[20] Alten B., Maia C., Afonso M.O., Campino L., Jiménez M., González E., Molina R., Bañuls A.L., Prudhomme J., Vergnes B. (2016). Seasonal Dynamics of Phlebotomine Sand Fly Species Proven Vectors of Mediterranean Leishmaniasis Caused by Leishmania Infantum. PLoS Negl. Trop. Dis..

[21] Dumitrache M.O., Nachum-Biala Y., Gilad M., Mircean V., Cazan C.D., Mihalca A.D., Baneth G. (2016). The Quest for Canine Leishmaniasis in Romania: The Presence of an Autochthonous Focus with Subclinical Infections in an Area Where Disease Occurred. Parasit. Vectors.

[22] Cazan C.D., Ionică A.M., Matei I.A., D’Amico G., Muñoz C., Berriatua E., Dumitrache M.O. (2020). Detection of Leishmania Infantum DNA and Antibodies against *Anaplasma* Spp., Borrelia Burgdorferi s.l. and Ehrlichia Canis in a Dog Kennel in South-Central Romania. Acta Vet. Scand..

[23] Cimpan A.A.D.I.P. (2019). Serological Study of Exposure to Leishmania in Dogs Living in Shelters in South-East Romania. Rev. Rom. Med. Vet..

[24] Mitková B., Hrazdilová K., D’Amico G., Duscher G.G., Suchentrunk F., Forejtek P., Gherman C.M., Matei I.A., Ionică A.M., Daskalaki A.A. (2017). Eurasian Golden Jackal as Host of Canine Vector-Borne Protists. Parasit. Vectors.

[25] (1956). Minculescu M Primul Focar de Leishmanioză Infantilă Identificat În România. Stud. Cercet. Inframicrobiol..

[26] Erscoiu S.V.C.F.S.C.E. (2014). Imported Visceral Leishmaniasis in Romania. Rom. J. Parasitol..

[27] Gogoaşe M.G., Teodorescu I., Preda C., Ionescu S.C. (2013). Two Case Reports on Visceral Leishmaniasis Diagnosed in Romania. Roum. Arch. Microbiol. Immunol..

[28] Carnielli J.B.T., Dave A., Romano A., Forrester S., de Faria P.R., Monti-Rocha R., Costa C.H.N., Dietze R., Graham I.A., Mottram J.C. (2022). 3′Nucleotidase/Nuclease Is Required for Leishmania Infantum Clinical Isolate Susceptibility to Miltefosine. EBioMedicine.

[29] Karmakar S., Volpedo G., Zhang W.-W., Lypaczewski P., Ismail N., Oliveira F., Oristian J., Meneses C., Gannavaram S., Kamhawi S. (2022). Centrin-Deficient Leishmania Mexicana Confers Protection against Old World Visceral Leishmaniasis. npj Vaccines.

